# Variations of the Candidate *SEZ6L2* Gene on Chromosome 16p11.2 in Patients with Autism Spectrum Disorders and in Human Populations

**DOI:** 10.1371/journal.pone.0017289

**Published:** 2011-03-04

**Authors:** Marina Konyukh, Richard Delorme, Pauline Chaste, Claire Leblond, Nathalie Lemière, Gudrun Nygren, Henrik Anckarsäter, Maria Rastam, Ola Ståhlberg, Frederique Amsellem, I. Carina Gillberg, Marie Christine Mouren-Simeoni, Evelyn Herbrecht, Fabien Fauchereau, Roberto Toro, Christopher Gillberg, Marion Leboyer, Thomas Bourgeron

**Affiliations:** 1 Human Genetics and Cognitive Functions, Institut Pasteur, Paris, France; 2 CNRS URA 2182 “Genes, synapses and cognition”, Institut Pasteur, Paris, France; 3 Service de Psychopathologie de l'Enfant et de l'Adolescent, Hôpital Robert Debré, Assistance Publique-Hôpitaux de Paris, Paris, France; 4 INSERM, U 995, IMRB, Department of Medical Genomic, Psychiatry Genetic team, Creteil, France; 5 Department of Child and Adolescent Psychiatry, Göteborg University, Göteborg, Sweden; 6 Institute of Clinical Sciences, Lund University, Malmö, Sweden; 7 Department of Clinical Sciences in Lund, Lund University, Lund, Sweden; 8 University Paris Est-Créteil, Faculty of Medicine, IFR10, Creteil, France; 9 AP-HP, Henri Mondor-Albert Chenevier Hospitals, Department of Psychiatry, Creteil, France; 10 Fondation FondaMental, French National Science Foundation, Créteil, France; 11 University Denis Diderot Paris 7, Paris, France; 12 Saint George's Hospital Medical School, London, United Kingdom; Seattle Children's Research Institute, United States of America

## Abstract

**Background:**

Autism spectrum disorders (ASD) are a group of severe childhood neurodevelopmental disorders with still unknown etiology. One of the most frequently reported associations is the presence of recurrent *de novo* or inherited microdeletions and microduplications on chromosome 16p11.2. The analysis of rare variations of 8 candidate genes among the 27 genes located in this region suggested *SEZ6L2* as a compelling candidate.

**Methodology/Principal Findings:**

We further explored the role of *SEZ6L2* variations by screening its coding part in a group of 452 individuals, including 170 patients with ASD and 282 individuals from different ethnic backgrounds of the Human Genome Diversity Panel (HGDP), complementing the previously reported screening. We detected 7 previously unidentified non-synonymous variations of *SEZ6L2* in ASD patients. We also identified 6 non-synonymous variations present only in HGDP. When we merged our results with the previously published, no enrichment of non-synonymous variation in *SEZ6L2* was observed in the ASD group compared with controls.

**Conclusions/Significance:**

Our results provide an extensive ascertainment of the genetic variability of *SEZ6L2* in human populations and do not support a major role for *SEZ6L2* sequence variations in the susceptibility to ASD.

## Introduction

The autism spectrum disorders (ASD) are characterised by impairments in reciprocal social communication, and repetitive, stereotyped and ritualistic verbal and non-verbal behaviours [1]. Beyond this unifying definition lies an extreme degree of clinical heterogeneity, ranging from profound to moderate impairments. ASD include autism, Asperger syndrome and pervasive developmental disorder not otherwise specified (PDD-NOS). The prevalence of ASD overall is about 1/100, but closer to 1/300 for typical autism [2]. Twin and family studies have conclusively described ASD as the most “genetic” of neuropsychiatric disorders, with concordance rates of 82–92% in monozygotic twins versus 1–10% in dizygotic twins, and a sibling recurrence risk of 6% [3], [4]. Several genes associated with ASD appear to be involved in synapse formation and/or maintenance, suggesting a common pathway in the susceptibility to these heterogeneous disorders [5], [6].

Chromosomal rearrangements have been recurrently associated with ASD. *De novo*, or inherited, submicroscopic microdeletion/duplication at the 16p11.2 chromosomal region are among the most frequently observed: 1% of ASD patients [7], [8], [9]. A meta-analysis and an additional screening provided support for the role of recurrent 16p11.2 deletions and duplications as a risk factor for ASD (deletions in 17/2172 ASD cases versus 9/28406 controls, P = 2.3×10^−13^; duplications in 10/2172 ASD cases versus 8/28406 controls, P = 1.9×10^−7^) [10]. Duplications of 16p11.2 have been identified in 0.3% of patients with schizophrenia (P = 4.8×10^−7^) and 0.12% of patients with bipolar disorders (P = 0.017), suggesting that 16p11.2 micro-rearrangements may be a vulnerability factor shared by patients with ASD, schizophrenia and bipolar disorders [10].

The critical 16p11.2 region spans 500–600 kb and is flanked by 147 kb highly similar (>99% homology) low copy repeats, that predispose this region to unequal crossing-over during meiosis. The region contains 27 genes that code for proteins, and apparently harbors no miRNAs. At least 17 of the genes are expressed in the brain and/or may play a role in neurodevelopment. To identify the causative genes for ASD, Kumar *et al.* (2009) screened for rare variations in 8 candidate genes that were selected based on their expression in the brain and function: *ALDOA*, *DOC2A*, *HIRIP3*, *MAPK3*, *MAZ*, *PPP4C*, *SEZ6L2* and *TAOK2* [11]. None of these genes showed a strongly significant association with ASD. However, a suggestive association was detected between the R386H non-synonymous variant in the *SEZ6L2* gene (seizure related 6 homolog (mouse)-like 2) and ASD (12/1106 ASD cases versus 3/1161 controls; P = 0.018). *SEZ6L2* is a compelling candidate gene for ASD due to the high level of expression in the brain and the strong homology of the protein with SRPX2 (Sushi-repeat-containing protein, X-linked), whose gene mutations causes epilepsy and language disorders [12]. Indeed, the prevalence of seizures in patients with ASD is between 5–38%, with the frequent observation of epileptiform activity, even without clinical epilepsy [13].

The aim of our study was to explore the genetic variability of *SEZ6L2* in an independent group of ASD patients. Since Kumar *et al.* (2009) had already sequenced a large sample of controls of European ancestry (n = 93–1731, depending on the exon), we chose to extend the mutation screening to other ethnic backgrounds from the Human Genome Diversity Panel (HGDP), to ascertain whether the variations identified in ASD are independent of the ethnic ancestry.

## Results and Discussion

**Figure 1:**
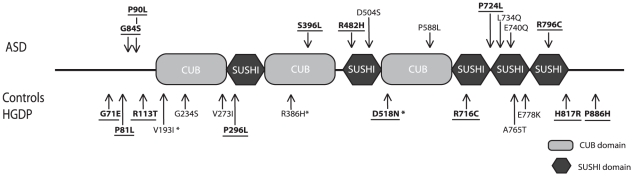
Protein localization of SEZ6L2 non-synonymous variations detected in patients with ASD, controls and HGDP samples. The changes predicted by PolyPhen-2 and/or SIFT as damaging for protein function, are indicated by underlined bold font. The variations found in both ASD and in control and/or HGDP groups are indicated with an asterisk.

We detected seven variations that are unique to ASD patients and six HGDP-specific ones. Furthermore, we found a single variation in both study groups (Table 1, Figure 1). When we merged our ASD screening results with those of Kumar *et al.* (2009), it could be concludes that ten non-synonymous variations were only detected in ASD patients, six variations were only observed in the control group and one was found in both groups (Table 1). Six of the 10 variations only reported in ASD (G84S, P90L, S396L, R485H, P724L and R796C) were predicted as probably damaging by the PolyPhen-2 (http://genetics.bwh.harvard.edu/pph/index.html) and/or SIFT (http://sift.jcvi.org) programs.

**Table 1:** Details and consequences of non-synonymous variations in the *SEZ6L2* gene identified in two independent sample set of patients with ASD, HGDP, and in ASD and control groups of Kumar *et al.* (2009).

Amino Acid Change	Functional prediction PolyPhen-2/SIFT	This study		Kumar *et al.* (2009)	
		ASD	HGDP	ASD	Controls
**ASD only**					
G84S	Benign/damaging	1/170^a^	0/282	0/527	0/278
P90L	Damaging/damaging	2/170^a^	0/282	0/527	0/278
S396L	Damaging/tolerated	0/170	0/282	1/1099	0/1152
R482H	Damaging/tolerated	1/170^a^	0/282	0/93	0/93
D504S	Benign/tolerated	1/170^a^	0/282	0/93	0/93
P588L	Benign/tolerated	0/170	0/282	1/527	0/278
P724L	Damaging/tolerated	0/170	0/282	1/527	0/554
L734Q	Benign/tolerated	0/170	0/282	1/527	0/554
E740Q	Benign/tolerated	1/170^a^	0/282	0/527	0/554
R796C	Damaging/tolerated	1/170^a^	0/282	0/527	0/554
**ASD and controls**					
V193I	Benign/tolerated	1/170^a^	0/282	0/527	2/278
R386H	Benign/tolerated	0/170	1/282^a^	12/1106	3/1161
D518N	Damaging/tolerated	4/170^a^	1/282^a^	0/93	0/93
**Controls only**					
G71E	Damaging/damaging	0/170	1/282^b^	0/527	0/278
P81L	Damaging/damaging	0/170	0/282	0/527	1/278
R113T	Damaging/tolerated	0/170	1/282^a^	0/527	0/278
G234S	Benign/tolerated	0/170	0/282	0/527	1/278
V273I	Benign/tolerated	0/170	2/282^b^	0/527	1/278
P296L	Benign/damaging	0/170	1/282	0/527	0/278
R716C	Damaging/damaging	0/170	0/282	0/527	1/554
A765T	Benign/tolerated	0/170	0/282	0/527	1/554
E778K	Benign/tolerated	0/170	1/282^b^	0/527	0/554
H817R	Damaging/tolerated	0/170	0/282	0/527	1/554
P886H	Damaging/tolerated	0/170	1/282^c^	0/527	0/278

a European/Middle East.

b Africa (Mandenka).

c Asia.

**Figure 2:**
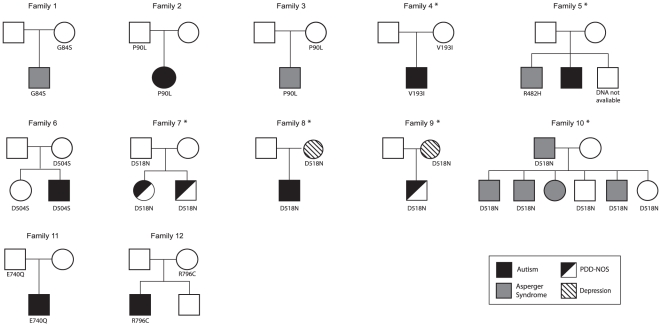
Pedigrees of families with rare non-synonymous variants of the *SEZ6L2* gene. Squares indicate males; circles indicate females. The black colour indicates individuals with a diagnosis of autism, grey indicates Asperger syndrome, a mix of black and white indicates PDD-NOS, and striped symbols represent depression. The variations found in control and/or HGDP groups are indicated with an asterisk.

In our cohort of patients, variations were inherited from healthy parents, with the exception of one that was transmitted by a father with Asperger syndrome (Figure 2). No significant difference between maternal (n = 7) and paternal (n = 5) transmission was observed. Variations we found in ASD samples were each sparsely distributed in only a small number of families and no common phenotype was observed among the carriers. For example, the G84S variation was maternally transmitted to a child with Asperger syndrome (Family 1). The P90L variation, predicted by both PolyPhen-2 and SIFT to be damaging, was found in 2 unrelated ASD families. In one family, the father transmitted this variation to his daughter who suffered from typical autism and intellectual disability (Family 2). This patient also carried a pericentric inversion on chromosome 9, which has no pathological consequences. The father's cousin is suspected to have Asperger syndrome, but his DNA was unavailable for this screening. In the second family, the mother transmitted the P90L variation to her son who had Asperger syndrome and obsessive–compulsive disorder (Family 3). A history of epilepsy was reported in neither family. In our study, the V193I variant was only observed in a single patient of Caucasian origin (Family 4). The same variation had already been detected in two Caucasian controls by Kumar *et al.* (2009) [11]. The R485H variation transmitted by a dyslexic father was observed in a child with Asperger syndrome and a high IQ (Family 5). The D504S variant was inherited from a father and was shared by two children, with and without autism (Family 6). The D518N variation was observed in 4 independent families with ASD, and in 1 subject in the HGDP (French Basque). In one family, the father transmitted the D518N variation to his two children, who both had PDD-NOS and encephalographic abnormalities (Family 7). In a second family, a boy with ASD and intellectual disability inherited the D518N variation from his mother, who was reported to suffer from a recurrent major depressive disorder (Family 8). In a third family, D518N was transmitted by another mother, with a major depressive disorder to her son, who had PDD-NOS and mild intellectual disability (Family 9). In a fourth large family, 3/4 children with Asperger syndrome carried the D518N variation, as did 2/2 unaffected sibs (Family 10). In this last case, this variation was inherited from the father diagnosed with Asperger syndrome. The E740Q substitution was observed in an autistic child and his healthy father (Family 11). The R796C variation was found in a single ASD family (Family 12), being transmitted from the mother to her son affected with autism, but not to her healthy son.

We also detected the two variations (R386H and E38E) that had previously been identified in patients with ASD by Kumar *et al.* (2009). The R386H variation was detected in a HGDP subject (Sardinian origin), but not in our ASD patient group. The synonymous E38E variation, that had previously been identified in a child with a 16p11.2 deletion and had been proposed as a risk allele, was detected in one patient (inherited from the mother). This child is mainly non-verbal and has a comorbid moderate intellectual deficit (IQ<50). We also identified this variation in two HGDP individuals (Russian and Orkadian origins). We did not find any of these variations in population samples from Africa, the Middle East and Asia.

Taken together, our results merged with those previously reported by Kumar *et al.* (2009) suggest that there is no significant enrichment of non-synonymous variations in the *SEZ6L2* gene in ASD compared with geographically matched European controls. In all but one family, variations were transmitted from unaffected parents and did not fully segregate with the disorder. In addition, in our patient group we did not detect the R386H variation that had previously been associated with ASD, nor did we observe *de novo* or non-sense mutations with putative functional impact. None of the variations detected in our ASD group overlapped with those reported by Kumar *et al.* (2009). Finally, when the predicted functional impact of the variations was considered, no enrichment in variations predicted to be damaging was observed in any of the three study groups: ASD (5/10), controls (3/6) and HGDP (3/5).

In conclusion, our screening of three supplemental groups of individuals from different ethnic backgrounds completes the determination of the genetic variability of *SEZ6L2*. However, the design of our study cannot formally exclude a causative role of the *SEZ6L2* gene.

One limitation of this study is the reduced power to detect association. Indeed, no alleles of frequency >1% have so far been definitively identified in ASD. Additionally, we lack functional assays to detect the impact of the detected variants on SEZ6L2 function. Here, we used PolyPhen-2 that has been reported to have a better prediction accuracy compared with the previous version [14]. However, we cannot exclude that functional approaches might yield a different result.

Finally, chromosomal rearrangements at 16p11.2 have been recurrently observed in several distinct developmental neuropsychiatric disorders [10], [15], [16]. It becomes evident that 16p11.2 duplications/deletions represent a significant genetic risk factor, but not sufficient by itself. Recent investigations suggest a “two-hit model” to develop the disease [15]. The decreasing cost of next-generation sequencing in the near future will shed light on the burden of mutations in coding parts and regulatory elements found in patients — not only for genes in the 16p11.2 region, but also for other genes that may act as additional hits.

## Materials and Methods

We sequenced all exons of the *SEZ6L2* gene in a sample of 452 individuals: 170 unrelated patients with ASD and 282 individuals from the Human Genome Diversity Panel (HGDP) [17]. Patients with ASD were recruited by the Paris Autism Research International Sibpair study at specialized clinical centres in France and Sweden. Diagnosis was based on clinical evaluation by experienced clinicians, using DSM-IV criteria, the Autism Diagnostic Interview-Revised (ADI-R) and the Autism Diagnostic Observation Schedule (ADOS). In Sweden, in some cases, the Diagnostic Interview for Social and Communication Disorders (DISCO-10) was applied instead of the ADI-R. Patients with Asperger syndrome were evaluated using the Asperger Syndrome Diagnostic Interview. Patients were included after a clinical and medical check-up with psychiatric and neuropsychological examination, standard karyotyping, fragile-X testing and brain imaging and EEG whenever possible. This study was approved by the local Institutional Review Board (IRB) and written inform consent was obtained from all participants of the study. The local IRB are the Comités de Protection des Personnes Île-de-France VI Sis Hôpital Pitié-Salpêtrière 75013 PARIS for France and the Sahlgrenska Academy Ethics committee, University of Gothenburg for Sweden. For all probands written inform consent was signed by the patients or parents or the legal representative.

The ASD sample (n = 170; 134 males, 36 females; 97 simplex and 73 multiplex families) included 154 unrelated patients with an autistic disorder and 11 with Asperger syndrome. Five individuals narrowly failed to reach the criteria for autistic disorders and were considered to have PDD-NOS. When a rare variation was identified, segregation was studied in first-degree relatives. The sample comprised 148 Caucasians, 6 Africans, 2 Asians, and 4 families of mixed ethnicity (self-report). In addition, we tested a sample of 282 HGDP individuals: 106 from Asia, 87 from sub-Saharian Africa and 89 from Europe and the Middle East (Table S1).

DNA was obtained from blood leukocytes or B-lymphoblastoid cell lines, and was extracted with phenol-chloroform. Mutations were screened by direct sequencing of the PCR products with specific primers (Table S2). We sequenced 17 coding exons of *SEZ6L2* (NM_201575.2) and one supplementary exon present in the other isoform (NM_012410.2) that had not previously been sequenced by Kumar *et al.* (2009). Amplification of 20 ng of DNA template was performed using specific primers. Two PCR protocols were used: (i) A standard protocol with the HotStart Taq polymerase (Qiagen) was used for all exons except exons 4 and 5: 95°C for 15 min, followed by 35 cycles of 95°C for 30 s, 57 to 62°C (depending on the Tm) for 30 s, 72°C for 30 s to 1 min (depending on the product size), with a final cycle at 72°C for 10 min; (ii) A Phusion High-Fidelity DNA polymerase (Finnzymes) standard protocol was used for exons 4 and 5: 98°C for 3 min followed by 35 cycles of 98°C for 10 s, 62°C for 25 s, and 72°C for 1 min, with a final cycle at 72°C for 10 min. Sequence analysis was performed by direct sequencing of the PCR products using the BigDye Terminator Cycle V3.1 Sequencing Kit, and an ABI PRISM genetic analyzer (Applied Biosystems). For all non-synonymous variations, the genotype was confirmed by sequencing an independent PCR product.

## Supporting Information

Table S1 (XLS)

Table S2 (XLS)
